# Orbital Cavernous Malformation Connected to Sporadic Cerebral Cavernous Malformations via a Developmental Venous Anomaly: A Report of Two Cases

**DOI:** 10.7759/cureus.104851

**Published:** 2026-03-08

**Authors:** Jorge Marcondes de Souza, Bernardo Lisboa Galvão Santos, Gabriel Verly, Nathalia Pentagna, Katia Carneiro, Luiz Celso Hygino da Cruz

**Affiliations:** 1 Neurosurgery Department, Federal University of Rio de Janeiro, Rio de Janeiro, BRA; 2 Institute of Biomedical Sciences, Federal University of Rio de Janeiro, Rio de Janeiro, BRA; 3 Neuroradiology Department, Hospital Pró-Cardíaco of Rio de Janeiro, Rio de Janeiro, BRA

**Keywords:** case report, cerebral cavernous malformation, developmental venous anomaly, orbital cavernous malformation, superior orbital fissure

## Abstract

Orbital cavernous venous malformations are isolated extradural vascular lesions with no known connection to intracranial pathology. We present two female patients with orbital cavernous malformations who were also found to harbor ipsilateral sporadic cerebral cavernous malformations. Using advanced 3T magnetic resonance imaging (MRI) sequences, including T1 3D dark blood, time of flight (TOF) 3D post-contrast, susceptibility-weighted imaging (SWI) with contrast enhancement, and 3D T1-weighted gradient echo (GRE) sequences, we identified in both cases an ectatic venous structure consistent with a developmental venous anomaly that traversed the superior orbital fissure, linking the orbital lesion to the intracranial venous system. Histopathological analysis of the surgically resected orbital lesion in the first patient confirmed the diagnosis of cavernous malformation. The information on the second patient relied solely on radiological analysis. Our findings indicate that a developmental venous anomaly may serve as a shared vascular conduit between orbital and intracranial cavernous malformations, challenging the view that orbital lesions arise in isolation. Screening for sporadic cerebral cavernous malformations should be considered in patients with orbital cavernous malformations when imaging demonstrates a developmental venous anomaly extending through the superior orbital fissure.

## Introduction

Orbital cavernous venous malformations (oCVMs), historically referred to as cavernous hemangioma, are the most common benign vascular lesions of the orbit. They are typically intraconal and retrobulbar, with a female predominance, suggesting a possible hormonal influence [1]. These lesions share histopathological features with cerebral cavernous malformations (CCMs), including endothelial-lined sinusoidal channels and occasional calcifications. However, the hemosiderin rim characteristic of cerebral lesions is less prominent in orbital ones [2]. Imaging differentiation can be challenging because the hallmark progressive nodular enhancement pattern overlaps with that of other vascular lesions [3].

Developmental venous anomalies (DVAs) are the most common intracranial vascular malformation, followed by cavernous malformations themselves [4]. DVAs are characterized by a “caput medusae” appearance, a fan-like arrangement of dilated medullary veins converging into a single transcortical or subependymal collector vein, representing an anomalous but functionally dominant venous drainage pathway. They are considered persistent variants of the embryonic venous drainage system and are generally benign [4]. Recent evidence suggests that DVAs act as a genetic primer for sporadic cerebral cavernous malformations (sCCMs), with an initial localized mutation preparing the ground for a second, disease-causing mutation [5]. Somatic gain-of-function mutations in *PIK3CA* and *MAP3K3* have been identified in both DVAs and their adjacent CCMs, suggesting that CCMs may arise from DVAs that have acquired additional mutations [5]. At 7-Tesla (7T) magnetic resonance imaging (MRI), the vast majority of sporadic CCMs are found adjacent to a DVA [6].

Although DVAs are recognized as drivers of sCCM formation within the brain, their potential to extend beyond the cranial compartment was not investigated. Two comprehensive reviews of DVA characteristics did not describe any connection with the orbit [4,7], and a classification of cavernous malformations by anatomical location explicitly categorized oCVMs as extradural lesions without DVAs [8]. Choudhri et al. reported a patient with co-occurrence of a CCM and an oCVM but did not identify a connecting vascular structure [2]. We present two patients in whom advanced MRI demonstrated a DVA connecting an oCVM to ipsilateral intracranial sCCMs through the superior orbital fissure (a narrow cleft between the orbital apex and the middle cranial fossa serving as a conduit for venous tributaries between the two compartments).

## Case presentation

Both patients were studied with a 3T MRI protocol (Skyra, Siemens) using T1 3D dark blood, time of flight (TOF) 3D post-contrast, susceptibility-weighted imaging (SWI) with contrast enhancement, and T1-weighted gradient echo (GRE) sequences, selected to maximize the detection of venous structures and cavernous malformations.

Case 1

A 35-year-old woman (currently aged 43-44) was referred to our institution for a large vascular lesion involving the left forehead skin, extracranial soft tissue, intracranial tissue, and retrobulbar space. The lesion presented as a single infiltrative process with both extracranial and intracranial components; histopathology confirmed that the intracranial component was cavernous rather than purely venous in nature, and it had resulted in left-sided vision loss. Initial imaging also revealed a sCCM in the left temporal ventricle, characterized by a heterogeneous "popcorn-like" signal on T1 and T2-weighted sequences with a surrounding hemosiderin rim, along with a dilated left basal vein of Rosenthal (BVR) consistent with venous engorgement. Underwent surgical resection of the orbital lesion via a transconjunctival approach in 2017 (age 35), with histopathological confirmation of a cavernous malformation [9]. She was subsequently followed at our outpatient clinic.

**Figure 1:**
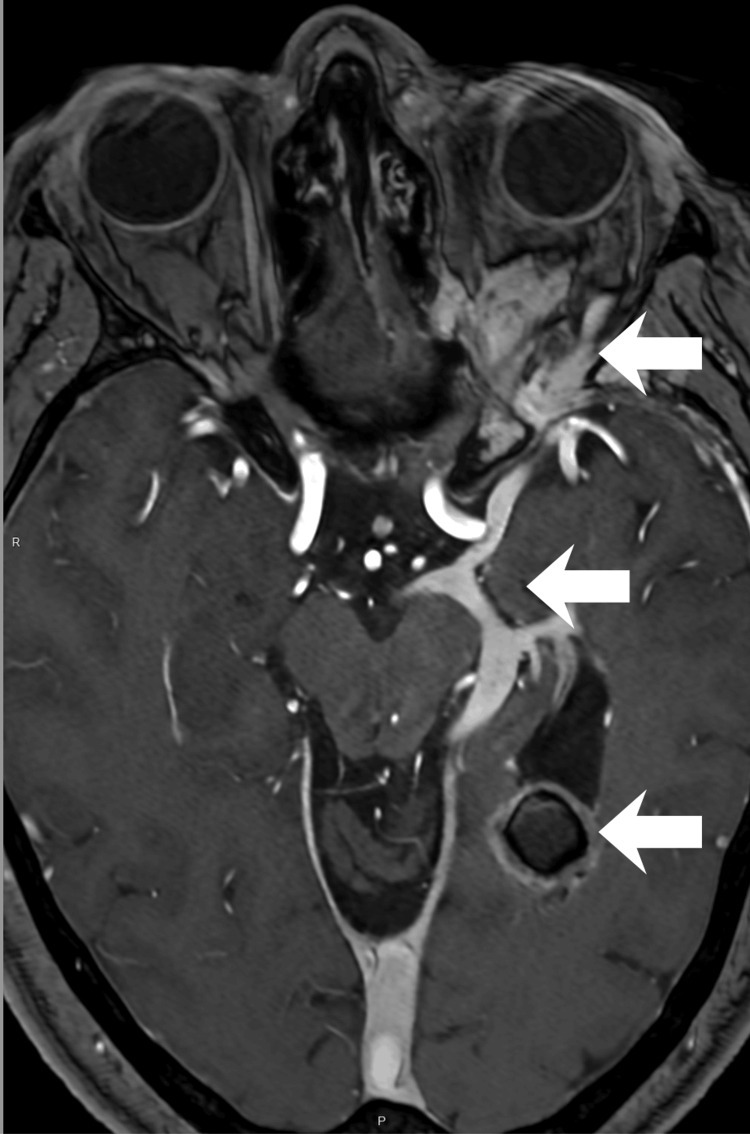
Orbital cavernous venous malformation and venous drainage MRI obtained six years after surgical resection of the primary orbital lesion showing a contrast-enhancing heterogeneous lesion in the posterior left orbit, associated with ectatic and tortuous vascular structures draining to the superficial medial cerebral veins through the superior orbital fissure, connecting to a dilated basal vein of Rosenthal and to the cavernous malformation in the left lateral ventricle. MRI: magnetic resonance imaging

**Figure 2:**
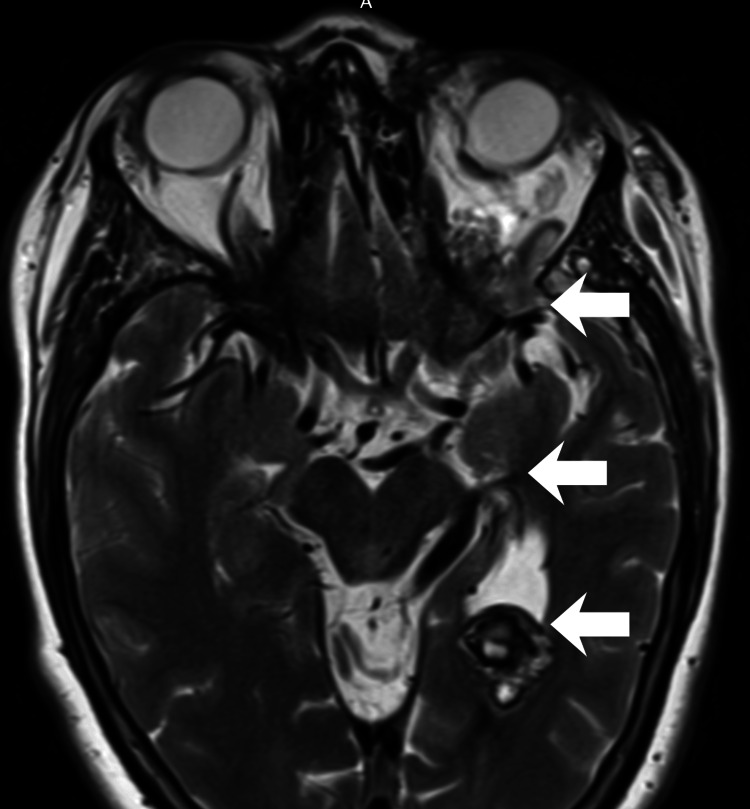
Deep venous connection and intraventricular cavernoma T2-weighted image demonstrating dilated superficial medial cerebral veins and basal vein of Rosenthal, connecting to a heterogeneous lesion with hemoglobin degradation products in the posterior aspect of the left lateral ventricle occipital horn, compatible with a cavernoma.

**Figure 3:**
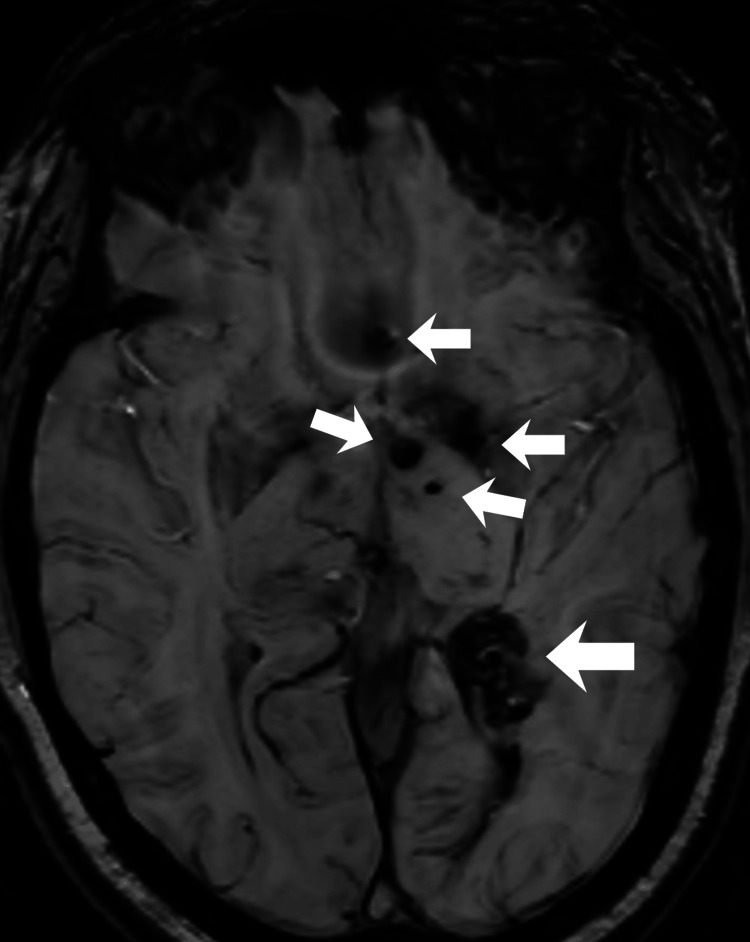
Multifocal supratentorial lesions Susceptibility-weighted imaging (SWI) sequence showing lesions in the left basal ganglia with imaging characteristics consistent with cavernomas.

After eight years of uneventful follow-up, she developed mild engorgement of the left upper eyelid at age 43-44, prompting reassessment by imaging. A follow-up MRI demonstrated a contrast-enhancing lesion in the posterior left orbit (Figure 1), connected to the intracranial venous system by an ectatic, tortuous vascular structure traversing the superior orbital fissure, displaying irregular caliber and serpiginous morphology on post-contrast sequences. This structure drained into the superficial medial cerebral veins and the BVR (Figure 2). Multiple additional sCCMs, beyond the previously known intraventricular lesion, were identified on SWI in the left basal ganglia (Figure 3), all ipsilateral to the orbital lesion. Interval development of new ectatic venous channels with abnormal contrast enhancement was observed, suggesting possible progression of the associated venous architecture over the eight-year period.

Case 2

**Figure 4:**
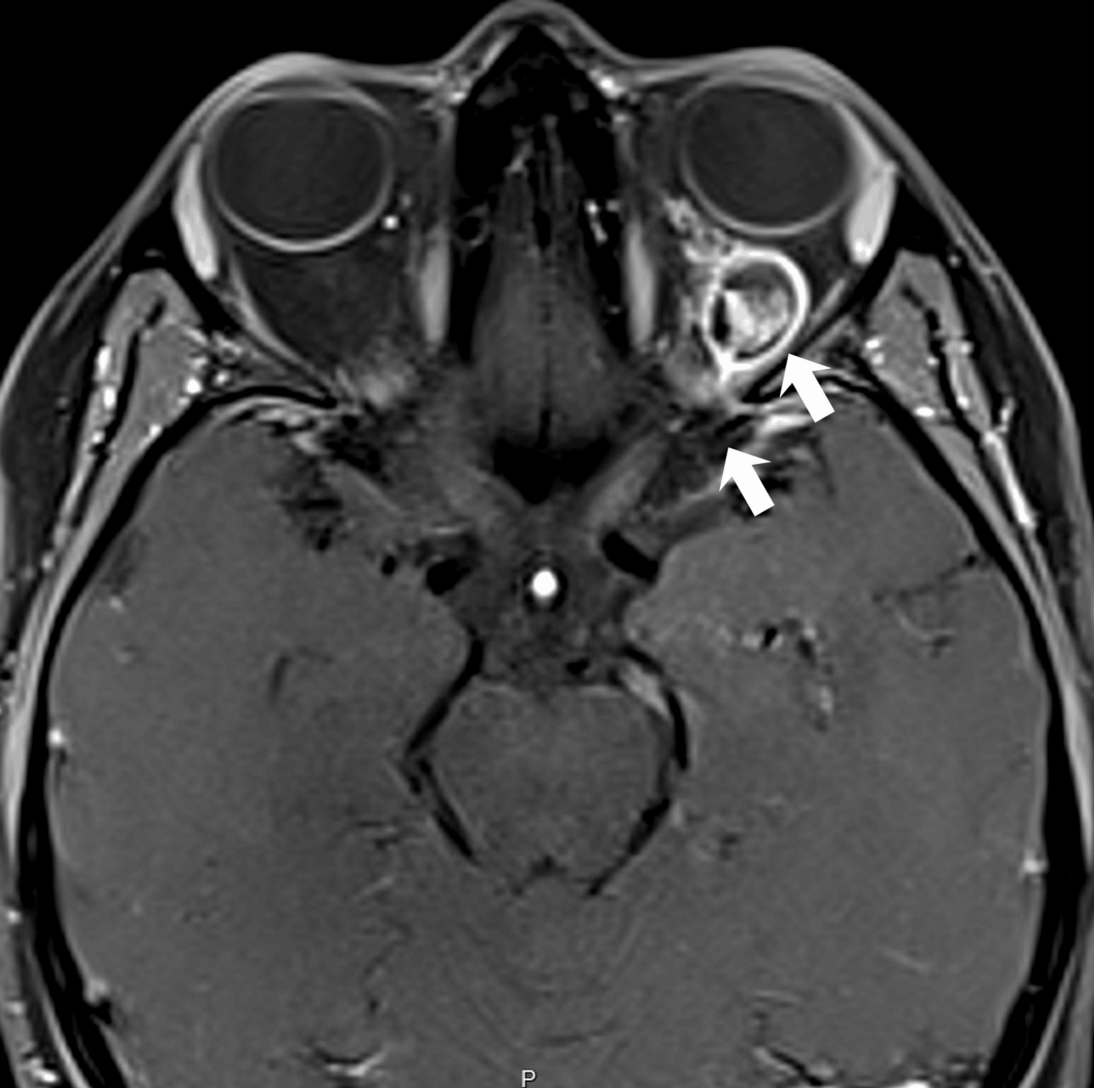
Retro-orbital cavernoma and venous extension 3D T1-weighted gradient echo (GRE) contrast-enhanced image of an intraconal, retro-orbital heterogeneous lesion containing hemoglobin degradation products, consistent with a cavernoma, associated with small ectatic vascular structures extending into the superficial medial cerebral veins through the superior orbital fissure.

**Figure 5:**
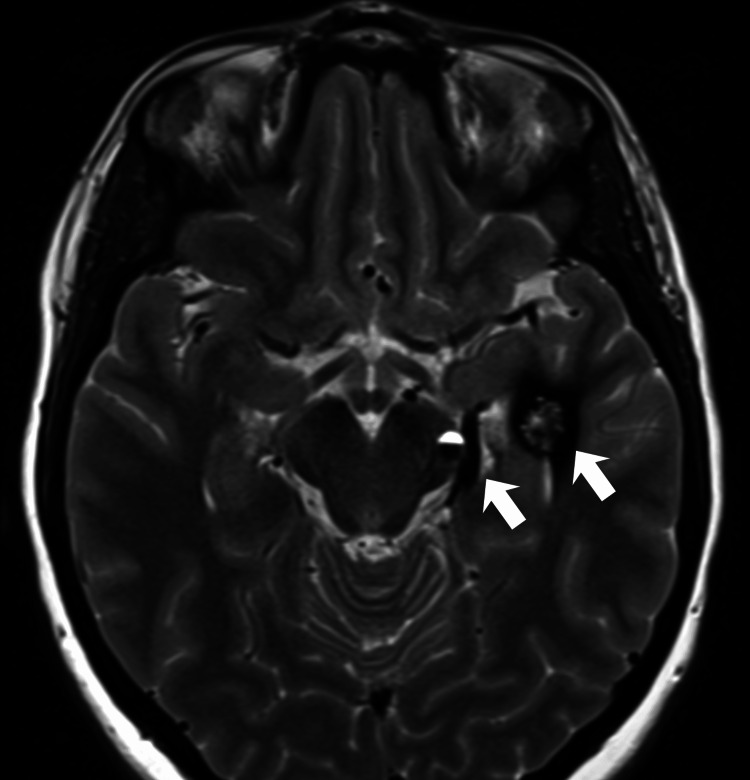
Temporal lobe cavernoma and associated DVA T2-weighted image showing a typical cavernous malformation in the temporal lobe, a dilated basal vein in the lateral basal cistern, and a mixed-signal brainstem lesion with hemorrhage. DVA: developmental venous anomalies

**Figure 6:**
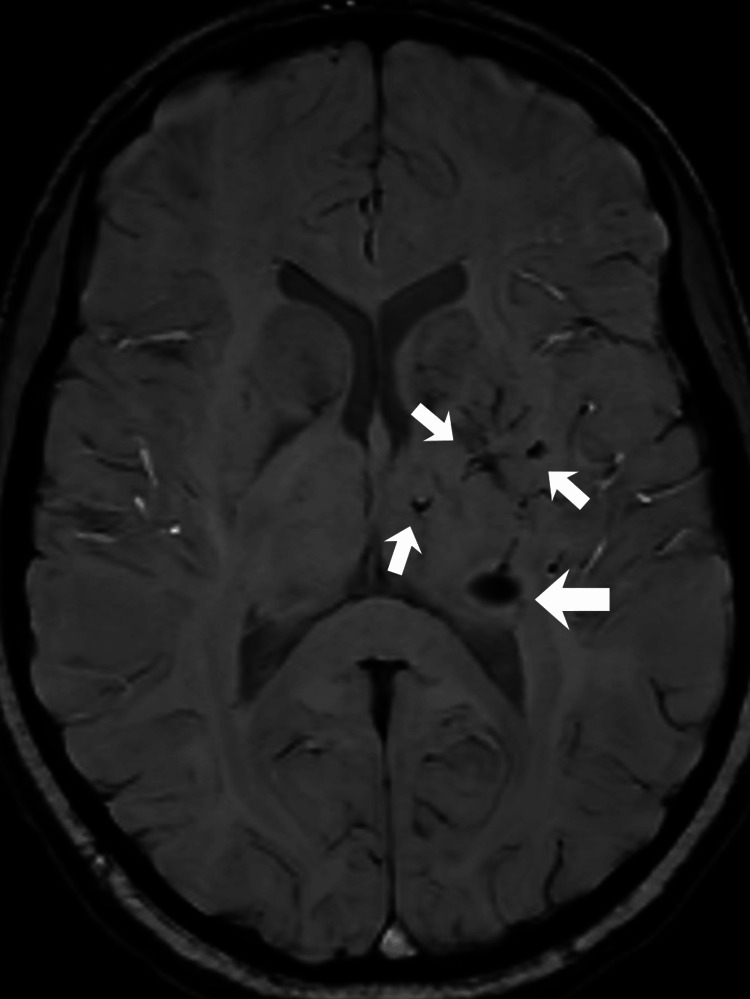
Thalamocapsular lesions and DVA SWI sequence demonstrating heterogeneous hypointense lesions in the lenticulocapsular region and posterolateral thalamus, indicative of cavernomas, accompanied by ectatic vascular structures suggestive of a developmental venous anomaly. DVA: developmental venous anomalies; SWI: susceptibility-weighted imaging

**Figure 7:**
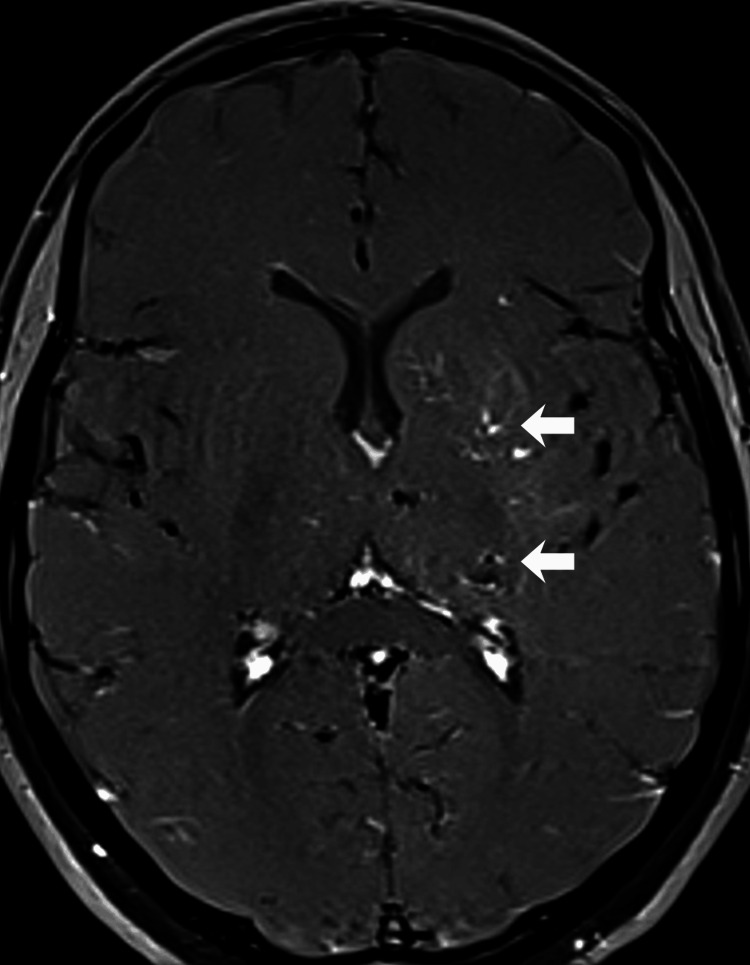
Deep venous vascularization Time of flight (TOF) 3D contrast-enhanced sequence showing extensive vascularization of the left deep cerebral hemisphere structures and a lesion in the posterior thalamic region.

A 25-year-old woman presented with exophthalmos after the recent use of levonorgestrel for emergency contraception. There was no visual impairment. MRI revealed an intraconal, retro-orbital heterogeneous lesion on T1-weighted sequences, displaying hemoglobin degradation products with T1 hyperintensity and susceptibility blooming on SWI, consistent with an orbital cavernous malformation. A small ectatic venous structure, consistent with a DVA, connected this lesion to the superficial medial cerebral veins through the superior orbital fissure (Figures 4, 5). Ipsilateral intracranial sCCMs were identified in the left brainstem surface, demonstrating T1 hyperintensity, fluid-fluid levels, susceptibility blooming on SWI, and perilesional edema consistent with recent hemorrhage, as well as in the lenticulocapsular region and the posterolateral thalamus (Figures 6, 7). All brain lesions were located in close proximity to the branches of a DVA displaying a "caput medusae" pattern, with multiple medullary veins radiating from a central collector vessel draining into a deep venous structure, a configuration associated with the development of multiple sCCMs. The patient remains asymptomatic with respect to left-sided vision. Despite the suggested surgical intervention for the orbital lesion, a conservative approach was elected in view of her asymptomatic status. She is currently under active clinical follow-up.

## Discussion

We describe two patients in whom advanced MRI demonstrated a DVA connecting an oCVM to multiple ipsilateral sCCMs through the superior orbital fissure. To the best of our knowledge, this vascular connection between orbital and intracranial cavernous malformations has not been previously reported. Choudhri et al. described the co-occurrence of a cerebral CM and an oCVM in a single patient, but no connecting DVA was identified, and the authors considered the association potentially coincidental [2]. Our cases differ in that a continuous venous structure was visualized linking the two compartments.

The role of DVAs as precursors to sCCMs is increasingly supported by genetic studies showing shared somatic *PIK3CA* and *MAP3K3* mutations in DVAs and their adjacent CCMs [5,10]. The abnormal hemodynamics and chronic venous congestion associated with DVAs are thought to create a microenvironment of inflammation and oxidative stress that promotes the formation of new cavernous lesions [4,6]. In our patients, the fact that all sCCMs were confined to a single hemisphere, ipsilateral to the orbital lesion and along the distribution of the DVA, supports a somatic rather than familial origin, and is consistent with the concept that these lesions arose in association with the DVA. The multiplicity pattern in our second case matches the Type III DVA subtype described by Bektas et al., which is most strongly associated with multiple sCCMs. Notably, that study did not screen for oCVMs [11].

Histopathological confirmation was obtained in Patient 1, whose orbital lesion was surgically resected and demonstrated typical features of a cavernous malformation [9]. Patient 2 was managed conservatively, given the absence of visual impairment. The imaging diagnosis in Patient 2 is based on the characteristic MRI appearance, including the presence of hemoglobin degradation products within the lesion [3,4].

Previous imaging studies of DVAs may have missed this orbital connection for several reasons. Brzegowy et al. reported DVAs in 7.46% of the adult population, with 4.14% having an associated sCCM. However, the majority of their images were acquired at 1.5T, which is less sensitive for detecting small DVAs [7]. Bektas et al. acknowledged that imaging variability, including inconsistent use of SWI versus GRE sequences and of 1.5T versus 3T equipment, may have led to underdetection of both DVA subtypes and sCCM multiplicity [11]. Neither study employed a protocol specifically designed to trace DVAs beyond the cranial compartment.

The hormonal dimension of these cases is noteworthy. The second patient developed exophthalmos after using levonorgestrel. Hormonal factors have been associated with the clinical course of oCVMs [1], and hormonal contraceptives have been linked to an increased risk of hemorrhage from CCMs [12]. This potential hormonal influence, combined with the radiologic progression observed in Patient 1, including interval development of additional vascular lesions over six years, underscores the importance of long-term imaging follow-up in patients with oCVMs associated with a DVA [13].

It is important to note that while the genetic evidence supporting a DVA-sCCM association is compelling [5,11,12], a definitive causal role for the DVA in generating the oCVM cannot be established from two cases alone. Genetic analysis of the lesions was not performed. Furthermore, while the imaging strongly suggests a continuous DVA connecting the orbital and intracranial compartments, alternative interpretations, such as independent co-occurrence, cannot be entirely excluded, particularly in Patient 1, where some intracranial lesions are relatively distant from the superior orbital fissure. However, the unilateral distribution of all lesions along the territory of a single DVA makes coincidental co-occurrence less likely.

We propose that oCVMs associated with a DVA extending through the superior orbital fissure may represent an underrecognized manifestation of the sporadic CCM-DVA complex. This phenomenon may have been previously misinterpreted as coincidental or overlooked entirely [14]. Although not hereditary, sporadic CCM may present with a multiple-lesion profile resembling familial disease [15]. In contrast to the diffusely distributed familial form, sporadic multiple CCMs tend to cluster along DVA branches [15,16].

## Conclusions

We report two cases involving a DVA traversing the superior orbital fissure connecting an oCVM to multiple ipsilateral intracranial sCCMs. This observation suggests that orbital CMs may occasionally represent the extracranial component of a sporadic CCM-DVA complex rather than isolated lesions. Advanced MRI protocols capable of detecting venous structures across the orbital-cranial boundary may be warranted in patients with oCVM, and screening for intracranial sCCMs may be considered when such a connection is identified. Further studies with larger cohorts and genetic analysis are needed to confirm and characterize this association.
